# An Assessment of the Population Dynamics and Evolutionary History of the Dingo

**DOI:** 10.1002/ece3.73152

**Published:** 2026-02-27

**Authors:** Carlo Pacioni, Danielle Stephens

**Affiliations:** ^1^ Arthur Rylah Institute for Environmental Research Heidelberg Victoria Australia; ^2^ Melbourne Veterinary, School, Faculty of Science University of Melbourne Werribee Victoria Australia; ^3^ Zoological Genetics Inglewood South Australia Australia

**Keywords:** *Canis dingo*, dingo, migration

## Abstract

Dingoes ( *Canis familiaris* ) are an iconic Australian species and the top land predator. Much interest exists in their radiation process and evolutionary history in Australia. Recent research indicated that two evolutionarily independent units exist and that detected effective population size changes are due to the active control of this species. However, these conclusions have been critiqued because they were not explicitly tested or because the model assumptions may not be met in dingoes. We set out to statistically test these hypotheses by comparing alternative migration models and carrying out demographic analyses. We conclude that there is strong statistical support for the existence of the two evolutionary units. However, the analysis carried out to estimate the time of the effective population size changes does not have the required power to conclusively demonstrate whether the current management is having an impact on dingo populations. Future studies and different approaches will be needed to test this hypothesis.

## Graphical abstract

Dingoes ( *Canis familiaris* ) are an iconic Australian species and the top land predator. Recent research indicated that two evolutionarily independent units exist and that the detected effective population size changes are due to the active control of this species. However, these conclusions have been critiqued by some because they were not explicitly tested or because the model assumptions may not be met in dingoes. We set out to statistically test these hypotheses by comparing alternative migration models and carrying out demographic analyses.

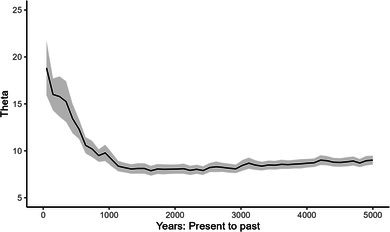

## 1 Introduction

Dingoes ( *Canis familiaris* ) are wild canids that occur throughout most of Australia. They are the largest land predator and their ecology and radiation processes are of great interest. Recent publications (Cairns and Wilton 2016; Cairns et al. 2017, 2018) have claimed that dingo populations have at least two evolutionarily separate populations (north west Australia, NW; and south east Australia, SE) or possibly three considering K'Gari (Fraser Island). Furthermore, Cairns et al. (2017) estimated demographic changes over time in these populations using a coalescent‐based approach. In that study, they detected an increase in the effective population size of the SE population and proposed two possible explanations: one where they suggest that it could have been a population expansion as a result of the thylacine extinction, or alternatively, that the high level of persecution of the south‐eastern population since European colonisation could have been responsible for the demographic change in this population.

However, these conclusions have been critiqued. The proposed NW‐SE evolutionary separation has been criticised based on the fact that the statistics used were inadequate (Jackson et al. 2017, 2019). These analyses, with the exception of the analyses of the mitochondrial DNA, aimed to detect population structure (which implies demographic separation), but, in itself, do not necessarily indicate that this separation is of evolutionary significance. Should the latter be the case, these populations would be expected to have been separated for a long enough time to allow for evolutionary changes. These could have started at the time of dingoes' colonisation of Australia, or potentially be reflective of a divergence that was already present in the founders of the Australian populations. On the contrary, demographic isolation caused by more recent events (e.g., European colonisation of the country) could be detectable even within a few generation times due to a shift in allele frequencies. Indeed, the independent evolutionary significance of these populations has not been statistically tested other than with mitochondrial DNA.

The coalescent‐based demographic analyses estimating effective population size used by Cairns et al. (2017) are also problematic because the model used to conduct these analyses was developed for panmictic populations (Drummond et al. 2005). If it is true that there are two separated populations with some level of migration between them (note that we refer here to migration within a population genetic rather than ecological context: the movement of alleles between populations), as the authors themselves specify (Cairns et al. 2017), then the published estimates could be biased (depending on the degree of migration present). Should the separation between the NW and SE populations be confirmed, a model that concurrently estimates each populations' size while considering migration could be a more appropriate method. Moreover, the genetic marker used (mtDNA) has a relatively slow mutation rate (in the order of 10^−8^/site/year) which may not be of sufficient resolution to infer population changes on a very recent time scale (i.e., the last 150 years).

We aimed to re‐analyse published data to explore whether historical and recent changes in dingo population dynamics claimed by the authors cited above are supported when statistically tested. Specifically, we propose to fit the data with four different coalescent‐based migration models (i.e., panmictic population, unidirectional migration and bidirectional migration) and identify the (statistically) most supported model. Within this approach, the support for a separate population system would indicate an evolutionary significance of each of its components. Furthermore, we intended to evaluate the possible difference between genetic markers (nuclear genes vs. maternally inherited genes) as there might also be sex‐biased dispersal in dingoes. Based on the most supported model, we then selected an adequate analytical framework to conduct demographic analysis (either with migration or for a panmictic population based on the results of the analyses suggested above) and repeated the demographic analysis from Cairns et al. (2017) using fast‐mutating genes (microsatellite loci), which could be more appropriate for investigating recent dynamics.

Conducting these evaluations matters for dingoes, as well as any other species for which population genetics is used to inform their management and conservation, because they provide confidence in the conclusions that were obtained, and can help in identifying the potential causes of the dynamics identified (e.g., by indicating the time of possible demographic changes) or alternatively can quantify the uncertainty around the results.

## 2 Methods

### 2.1 Datasets

Full mitochondrial sequences (*n* = 25) were obtained from (Cairns and Wilton 2016). We did not consider the publicly available mtDNA data from (Cairns et al. 2017) because these were generated by selecting mtDNA regions that would maximise the difference between the two populations (NW and SE), so we considered these data potentially biased for our purposes. We obtained sequence data (*n* = 25) from nuclear genes published by Cairns and Wilton (2016) and Single Nucleotide Polymorphism (SNP) data (*n* = 25) from Cairns et al. (2018). However, upon downloading the sequence data from nuclear genes published by Cairns and Wilton (2016), we realised that these data could not be used because polymorphism was reported with ambiguity codes making it impossible to reconstruct the phased alleles needed for our analyses. Hence, this dataset was not included in our analysis. Lastly, we obtained microsatellite data from Stephens et al. (2015). Most of the samples used by Cairns and Wilton (2016), Cairns et al. (2017), and Cairns et al. (2018) were also genotyped by Stephens et al. (2015), so these were included in the microsatellite datasets. However, we considered it necessary to increase the sample size since the microsatellite data were from a limited number of loci (23 loci). Hence, we randomly selected additional pure or probable pure dingoes (as defined by Stephens et al. 2015 using the 3Q method and reported in their supplementary material. This corresponds to a > 80% dingo ancestry profile based on Structure analysis) from each region, giving priority to individuals that were completely genotyped. The final microsatellite dataset included a total of 63 individuals (23 from the Alpine region, 12 from the Simpson desert, 14 from the Gibson desert and 15 from the Kimberley).

### 2.2 Data Analysis

Sequence data (mtDNA) were visualised and converted into relevant formats for analysis in Geneious (Kearse et al. 2012). SNP data were imported in Plink 1.07 (Purcell et al. 2007) and filtered as reported by Cairns et al. (2018). However, after the filtering steps, we had > 4000 loci removed compared to what was reported by Cairns et al. (2018). We noted that Cairns et al. (2018) used Plink 1.6, which was not equipped to handle the different number of chromosomes that dogs have compared to humans, and several loci were mapped to different chromosomes compared to our dataset (with no fault of the authors), which we assumed caused the mismatch between the published and our dataset. To ensure that the loci were mapped to the correct chromosomes, we used the manifesto released by Illumina with the list of the SNP identifiers and their mapping to select only autosomal loci. We then refined data by filtering individuals that had > 90% loci genotyped, removing loci with allele frequencies < 5% and only keeping loci with a 100% genotyping success rate. That left 43,901 loci suitable for analysis. We ran an Admixture (Alexander et al. 2009) analysis with *K* = 3 to confirm that we could replicate Cairns et al. (2018) population structure results.

We used the software Migrate‐n (Beerli 2006, v3.7.2 for sequence data and v5.0.4 for microsatellite and SNP data) to compare migration models after grouping the data based on the geographical location of sampling. Because of Migrate‐n limitation, a random selection of 5000 SNP data was used for this analysis. We started by implementing three population migration models (north west Australia, NW; south east Australia, SW and K'Gari). Because these analyses would not converge, we simplified the model by removing animals sampled in K'Gari and focused on the separation between NW and SE populations. We compared the following migration models: two populations with bi‐directional migration (Full migration); two populations with migration from the north west to the south east only (NW2SE); two populations with migration from the south east to the north west only (SE2NW); and one panmictic population where the NW and SE populations were effectively merged. Input files for Migrate‐n for the microsatellite data were generated with the software Create (Coombs et al. 2008). At least two independent runs were carried out with different MCMC lengths and analysis was considered converged when the results and the posterior distribution were approximately equal. Otherwise, we progressively increased the length of the burnin and the MCMC until the model reached convergence. Model selection was carried out by comparing the log Bayes factor calculated with the Bezier marginal likelihoods obtained by thermodynamic integration (Beerli and Palczewski 2010). Using the model with the highest log marginal likelihood as the reference model, a log Bayes factor < −2 is considered in support of the model, < −6 strongly in support of the model (Kass and Raftery 1995).

Coalescent‐based analyses to evaluate population size changes over time were conducted by constructing a Bayesian Skyline Plot in a final analysis with Migrate‐n and post‐processing outputs with the R package mtraceR (Pacioni et al. 2015). Migrate‐n divides time into equal intervals (epochs) that are set by the user. Due to the different mutation rate between the genetic markers, we used ‘intervals’ equal to 0.2 for SNPs and 0.011 for microsatellites, where the intervals are the number of generations scaled for the mutation rate. The effective population size is estimated for each of these intervals using the information contributed by the coalescent events within each interval. To convert the x‐axis (time) to years, we used a mean mutation rate of 10^−8^ for SNPs (Skoglund et al. 2015) and 5 × 10^−4^, which is a commonly used mean mutation rate for microsatellites (Garza and Williamson 2001; Sacks et al. 2013) and a generation time of 4.5 years (Sacks et al. 2013; Woinarski et al. 2014; Ballard et al. 2018). To verify the previously reported generation time estimates (4–5 years), we used the software AgeNe (Waples et al. 2011), which computes the generation time using age class survival and reproductive rates. We used age‐specific survival rates from Pacioni et al. (2018); and Pacioni et al. (2020), and set maximum age as the age at which the probability of survival was < 0.5%. Reproductive rates were calculated from Pacioni et al. (2020) simulated data from the scenarios where lethal control was not implemented. This is because, in evolutionary terms, lethal controls have been applied only for a small window of time and it was considered negligible when computing the average generation time. Two possible scenarios were considered when calculating generation time, one where the birth rates were constant until the last year of the animal's life, and one where these would be halved when the female was 8 years old and then zero once the female was 10 years old or older.

Given the contrasting results that we obtained in the migration model comparison between different genetic markers, we decided to further investigate the population structure to evaluate whether it was possible to detect patterns of gene flows between populations. To this end, we initially used Admixture (Alexander et al. 2009) with the SNP dataset. We conducted 10 runs for each *K* value (1 to 10) with 10 and 20 fold cross‐validation and considered the runs converged when the log‐likelihood change was < 0.1. We monitored the cross‐validation error to evaluate the most supported number of genetic clusters (*K*). To have more flexibility and test the effect of different priors, we then used the software Structure (Pritchard et al. 2000; Falush et al. 2003) using the subset of 5000 SNPs used for Migrate‐n analyses, for computational efficiency. We initially fixed *K* = 1 and estimated the value for the parameter *λ* using the default prior. This parameter controls the allele frequencies and it is estimated by fixing *K* = 1 because the analysis will incur identifiability issues with other parameters in the model as detailed by Pritchard et al. (2010). Once *λ* was estimated, it was fixed to its mean value in successive analyses and we ran the correlated and uncorrelated allele frequency models allowing independent α values (the admixture parameter) for each genetic cluster, using a uniform prior between 0 and 10. Suitable *K* values were considered by inspecting the mean log‐likelihood and using the Evanno et al. (2005) method (if *K* = 1 could be discounted when inspecting the log likelihood).

To further investigate the migration rate between populations, we used the model developed by (Wilson and Rannala 2003) using the software BA3‐SNPs for SNP data (Mussmann et al. 2019) and BA3 (Wilson and Rannala 2003) for microsatellite data. BA3‐SNPs implements the same model as BA3, but with a few coding modifications to allow the handling of more loci.

## 3 Results

### 3.1 Migration Model Comparison

As mentioned above, the three‐population model analysis did not converge, so we report here only results for the two‐population model (north west Australia, NW; south east Australia, SE).

The most supported model by the mtDNA data was the panmictic model, with a 76% probability, the unidirectional migration model from the NW to the SE population with 17% and the full migration model with approximately 7% probability. On the contrary, SNP data strongly supported the full migration model (100% probability). Lastly, the microsatellite data strongly supported the unidirectional migration model SE to NW (100% probability, Table 1).

**TABLE 1:** Comparison of marginal likelihood obtained with thermodynamic integration and Bezier approximation from the software Migrate‐n.

Genetic marker	Model	Log marginal likelihood	Log Bayes factor	Rank	Model probability
mtDNA	Full model	−22,582.18	−2.42854	3	0.067
	NW → SE	−22,581.71	−1.491252	2	0.171
	SE → NW	−22,585.24	−8.555256	4	0
	Panmictic	−22,580.96	0	1	0.761
SNPs	Full model	−115,113	0	1	1
	NW → SE	−119,469.9	−8713.76	2	0
	SE → NW	−120,330.8	−10,435.52	3	0
	Panmictic	−123,614.5	−17,002.98	4	0
msats	Full model	−67,271.44	−96,267.5	4	0
	NW → SE	−20,649.94	−3024.5	2	0
	SE → NW	−19,137.69	0	1	1
	Panmictic	−22,304.87	−6334.36	3	0

*Note:* Full model: two populations with bi‐directional migration; NW → SE: two populations with NW to SE migration only; SE → NW: two populations with SE to NW migration only; Panmictic: one population.

### 3.2 Demographic Analyses

**FIGURE 1:**
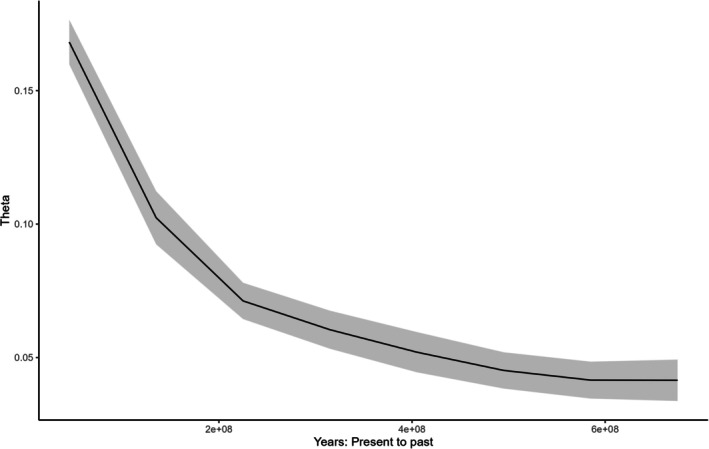
Demographic reconstruction through time for the SE population based on SNP data. Solid line: Median parameter estimates. Shaded area 1.96 × standard deviation. Theta = 4*μN*_e_ where *μ* is the mutation rate and *N*_e_ is the effective population size.

**FIGURE 2:**
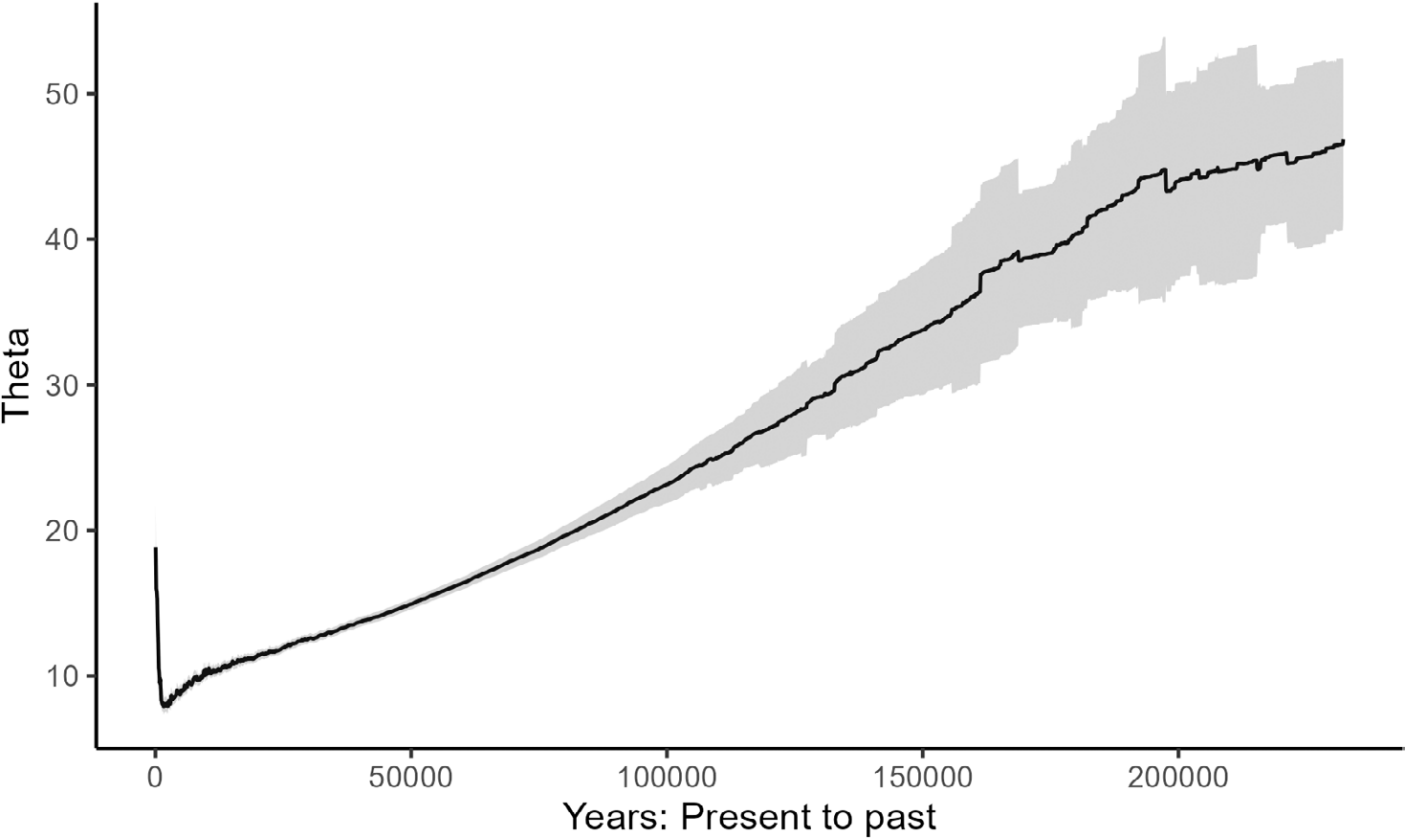
Demographic reconstruction through time for the SE population based on microsatellite data. Solid line: Median parameter estimates. Shaded area 1.96 × standard deviation. Theta = 4*μN*_e_ where *μ* is the mutation rate and *N*_e_ is the effective population size.

**FIGURE 3:**
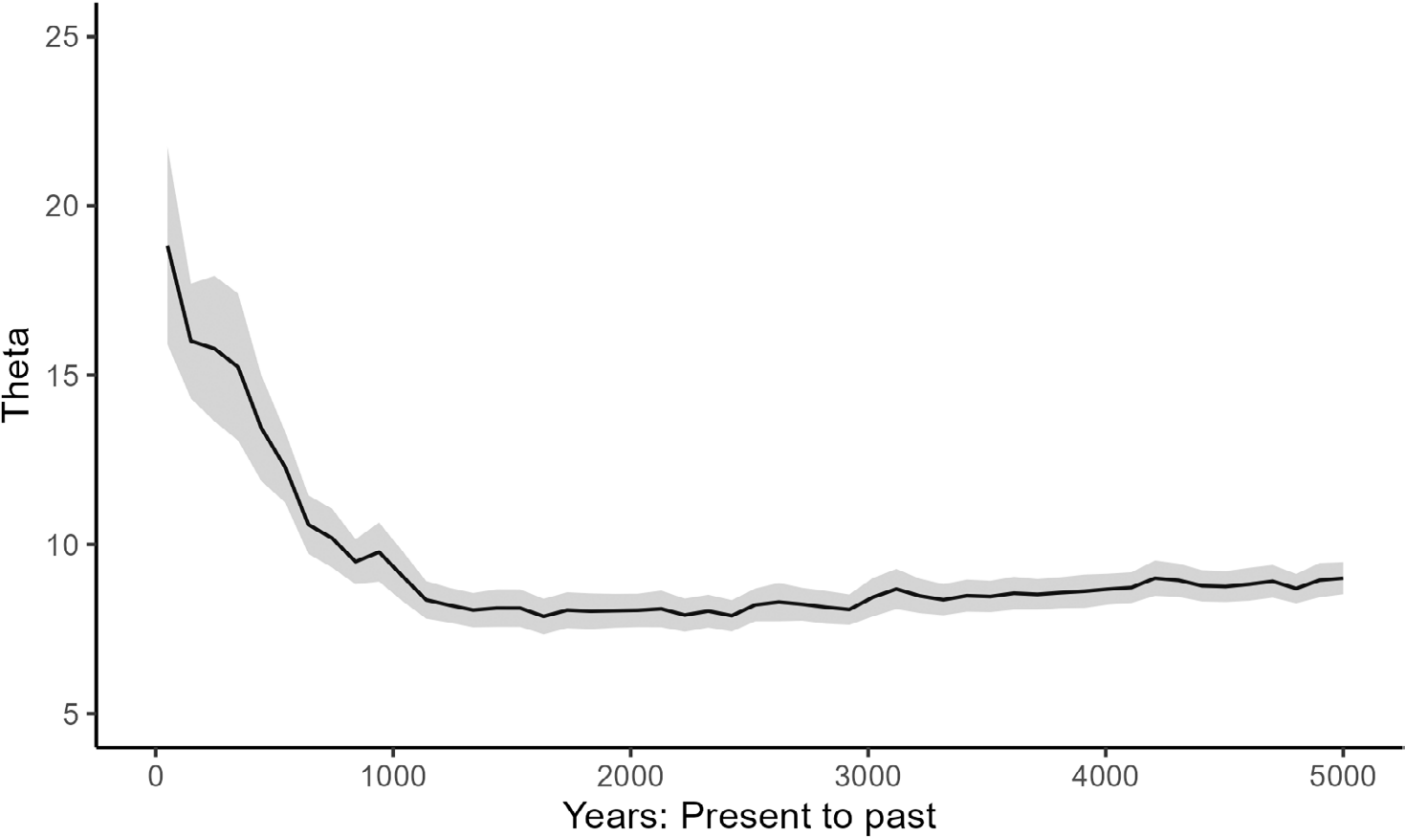
Demographic reconstruction through time for the SE population based on microsatellite data, limited between present‐day and 5000 years ago. Solid line: Median parameter estimates. Shaded area 1.96 × standard deviation. Theta = 4*μN*_e_ where *μ* is the mutation rate and *N*_e_ is the effective population size.

**FIGURE 4:**
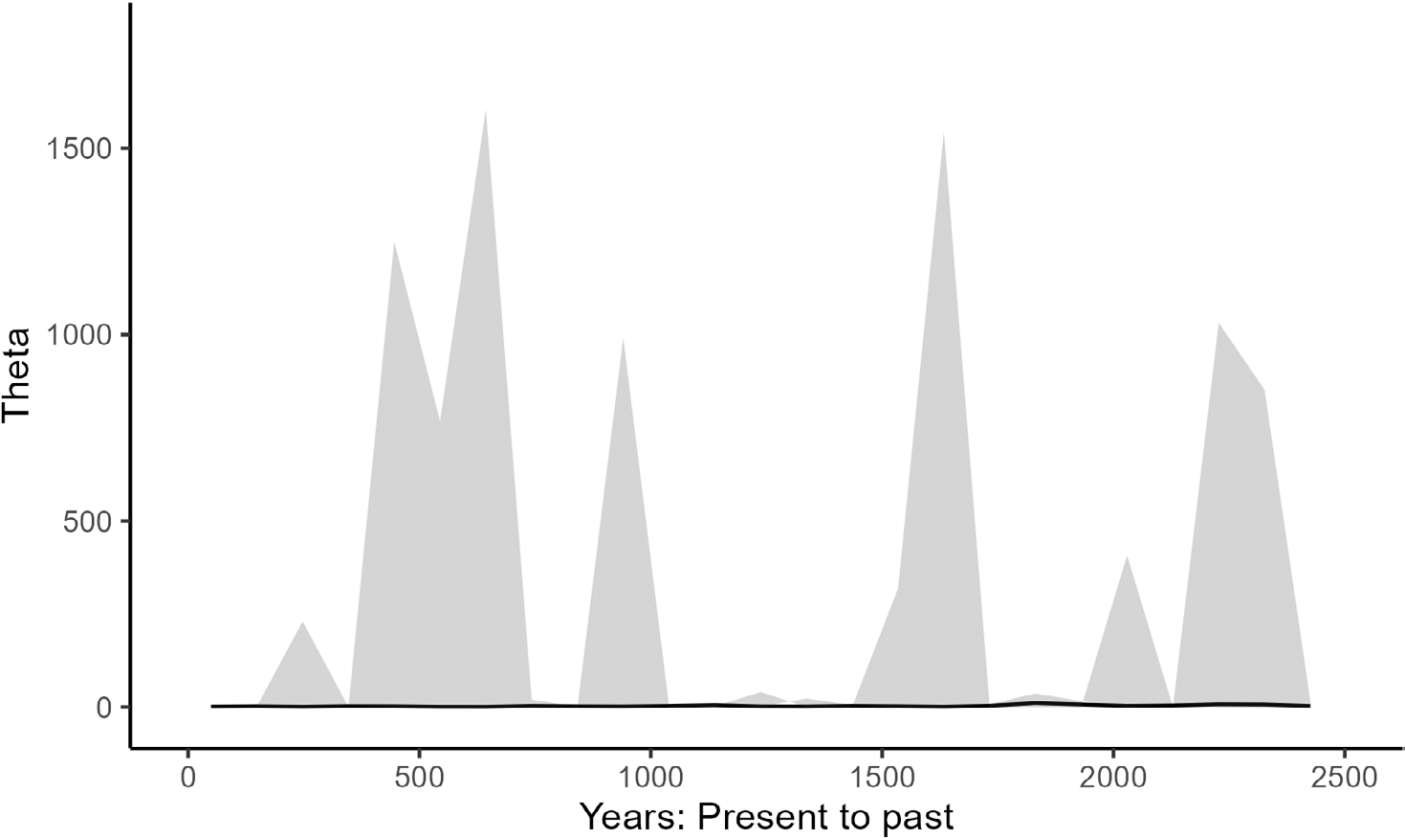
Demographic reconstruction through time for the NW population based on microsatellite data, limited for clarity between present‐day and 2500 years ago. Solid line: Median parameter estimates. Shaded area 1.96 × standard deviation. Theta = 4*μN*_e_ where *μ* is the mutation rate and *N*_e_ is the effective population size.

Generation time was estimated at 4.5 and 5 years, which is consistent with what is reported in the literature (Sacks et al. 2013; Woinarski et al. 2014; Ballard et al. 2018), so we used 4.5 years in our calculations to convert the mutation rate into years. Bayesian Skyline Plot generated with the SNP data was very similar for both populations (NW and SE). They both had a gradual increase in the effective population size starting from 2 × 10^8^ years ago that continued until the most recent epoch (For simplicity we only report here the SE, Figure 1). Owing to their faster mutation rate, microsatellite data inferred demographic changes for much more recent times. In fact, the most recent epoch with SNP data encompasses the last few tens of millions of years, while with microsatellite data it is about 100 years. The demographic reconstruction of the SE population suggested a decline from 2 × 10^5^ years ago until about 1000 years ago (Figure 2), after which the population began increasing in size (Figure 3). Demographic reconstruction for the NW population suggests a constant population size, although there is a high degree of uncertainty around the estimated parameter values (Figure 4). Indeed, the number of coalescent events within each epoch is fairly limited, and < 500 after the second epoch. This suggests that the information content in the data for this population is fairly limited.

### 3.3 Population Structure Analysis

#### 3.3.1 Admixture

**FIGURE 5:**
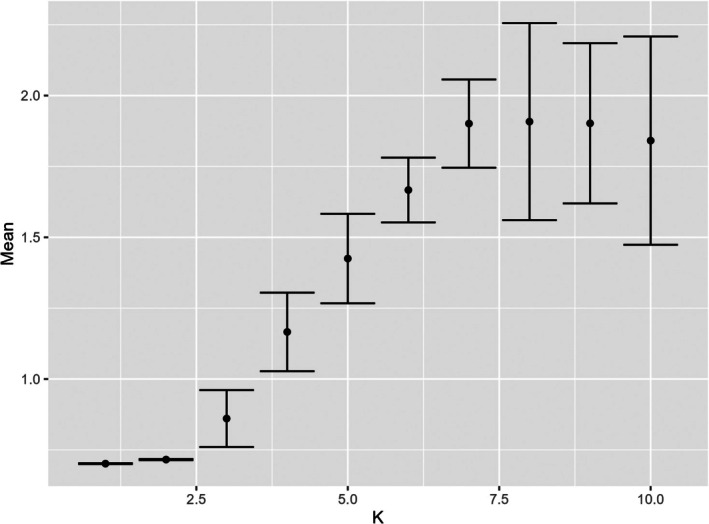
Cross‐validation errors (*y*‐axis, dot: Mean, error bars: Standard deviation) as a function of *K* values computed by Admixture (Alexander et al. 2009) with the SNP dataset and 20 fold cross‐validation. Convergence was declared when the log‐likelihood change was < 0.1.

Admixture analysis of the SNP dataset suggested *K* = 1 as the most supported *K* value (Figure 5). *K* = 2 had only a marginally higher cross‐validation error. Cross‐validation errors for *K* = 1 and *K* = 2 had very limited variability (measured as standard deviation), while from *K* = 3 this increased substantially (Figure 5). When considering *K* = 2, the individuals sampled from the Alpine region were all grouped in a separate genetic cluster from all the other sampling locations.

#### 3.3.2 Structure—Correlated Allele Frequencies Model

**FIGURE 6:**
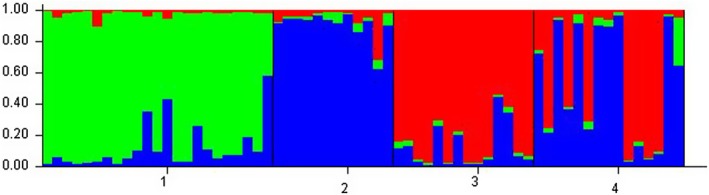
Bar plot of the proportion of membership of each individual (each column in the plot) obtained with Structure when using microsatellite data and *K* = 3. Thin black vertical lines mark the separation between sampling locations (1: Alpine region, 2: Simpson desert, 3: Gibson desert, 4: Kimberly region).

Structure analysis suggested *K* = 2 for both SNPs and microsatellite data when considering the correlated allele frequencies model and using Evanno's method. Inspecting the log‐likelihood profile, SNPs data suggested that *K* = 2 and *K* = 3 were possible, however the proportion of membership for each individual sampled in the Simpson desert was very close to 0.5 when *K* = 3, which is an informal pointer of model overfitting. The log‐likelihood profile for the microsatellite data analysis with this model (i.e., correlated allele frequencies model) suggested *K* = 3, with individuals from the Alpine region, the Simpson desert and Gibson desert each clustering separately, and individuals from the Kimberley being assigned (almost entirely) to either the Simpson desert or the Gibson desert (Figure 6).

#### 3.3.3 Structure—Uncorrelated Allele Frequencies Model

For both datasets (SNPs and microsatellites), and both methods (i.e., log‐likelihood profile inspection and Evanno's), our analysis suggested *K* = 2 when the uncorrelated allele frequencies model was used.

When *K* = 2 was considered, the individuals sampled from the Alpine region were all grouped in a separate genetic cluster from all the other sampling locations, with the exception of the microsatellite data analysis where the individuals sampled from the Alpine region and the Simpson desert were grouped together in a separate genetic cluster from all the other sampling locations.

## 4 Discussion

We set out to further explore the results presented in the recent literature on dingo genetic population structure and demographic dynamics (Cairns and Wilton 2016; Cairns et al. 2017, 2018). Our results are broadly supportive of the results previously reported; however, our analyses highlighted some differences and important characteristics of the different datasets.

The most supported migration model by the mtDNA data analysis was the panmictic model, although with only a 76% probability. It is well known that estimation of migration rates is challenging and the use of one locus only is not optimal for this purpose. Indeed, the same data supported migration models with the two populations separated with > 20% probability. Specifically for dingoes, this inference based on mtDNA is further complicated by the fact that genetic variability is limited and the dataset we analysed had a small sample size; hence, the data are likely not to contain sufficient information to support more complex models. The limited sample size is a common problem in many of the analyses we conducted; however, it was our intention to use comparable datasets (as much as possible) with previous research for the aims of this study. While for the SNP datasets the large number of loci should, in theory, compensate for the small sample size, with the mtDNA data interpretation of these results should be careful.

The fact that coalescent‐based analyses with both SNP and microsatellite loci suggest a two‐population model, even though each supports a different migration model, indicates that this separation has evolutionary importance. The demographic analysis using SNP suggested a similar history for both populations with a continuous increase in population size. Based on our assumption of an average mutation rate for this genetic marker of 10^−8^ (Zhang et al. 2025), we inferred that these changes occurred before the arrival of the dingo in Australia, and are likely related to the (ancestral) species' expansion. On the contrary, microsatellite data showed different demographic histories for the two populations. It is likely that the difference in the two analyses stems from the different evolutionary timeframes that data provide information on. As microsatellite data have a much faster mutation rate, they are better suited to infer more recent population size changes (tens to hundreds of generations), while SNP data carry over information from events deeper in time. It is also important to point out that, while Migrate‐n offers the advantage of considering migration in its estimates, the estimation of the time of the detected population changes is somewhat more rudimentary compared to other analytical frameworks. For example, in Migrate‐n analyses, time is divided into equal epochs without taking into account the information content provided by the data within each epoch, so depending on the datasets, Migrate‐n estimation of the timing of population size changes can be coarse. On the contrary, BEAST (Drummond et al. 2012), which has a much more advanced implementation of Bayesian Skyline Plot analysis (Drummond et al. 2005) and was used by Cairns et al. (2017), dynamically estimates the contribution of the different grouping of events, often fully exploiting the information contained in the data (however, other limitations of the method implemented in BEAST should not be forgotten. In our context, the fact that the analysis assumes a panmictic population is potentially particularly relevant). In our microsatellite analysis, we were able to greatly reduce the window size of the epochs as the information content (estimated as the number of coalescent events within each epoch) for the SE was relatively high (in the order of the thousands), and we obtained a relatively high resolution.

It is also interesting to note that the results from the microsatellite analysis are strongly in agreement with the mtDNA BSP analysis from Cairns et al. (2017). It is possible that despite not taking into account migration, BEAST analyses were not strongly biased because migration rates were relatively low. Both analyses (mtDNA by Cairns et al. 2017 and microsatellite data presented here) indicated that the timing of the population size change in the SE population started about 1000 or more years ago. That is, before the European arrival in Australia, and it is unlikely, contrary to what was suggested by Cairns et al. (2017), that these demographic changes were therefore caused by recent management of the species, and are more likely to be related to environmental, pre‐European anthropogenic, or ecological changes.

Interestingly, population structure analysis with Admixture supported a panmictic population once the K'Gari population was removed from the dataset. In that analysis however, *K* = 2 also obtained a low cross‐validation error and it is possible that other factors are at play. In fact, when repeating the analysis with Structure, which allows a more refined fine‐tuning of the priors, the underlying population structure (i.e., *K* > 1) became evident. The analysis with microsatellite data supported this result and possibly suggested that additional structuring is possible. Indeed, we would argue that with a more exhaustive geographical coverage, the complexity of the dingo population structure is likely to be further demonstrated. It is important to note that structure analysis conducted here aims to identify groups of individuals whose allele frequencies are in Hardy–Weinberg equilibrium, so does not provide information on the evolutionary history of the populations but rather provides an indication of the demographic (reproductive) independence of these.

In conclusion, we were able to confirm that the separation between the NW and SE evolutionary significant units suggested by Cairns et al. (2017), Cairns et al. (2018) and Cairns and Wilton (2016) is statistically supported. Moreover, the demographic changes through time while explicitly considering migration are also consistent with previously reported results. However, even when using fast‐mutating genetic markers, the timing of these demographic changes, estimated to have started about 1000 years ago, is not consistent with the suggestion that current lethal control (which was carried out only in the last 100 or so years) is the likely cause of these changes. This is not to say that current management might not affect the genetic make‐up of dingo populations, but it is not evident in these analyses as previously suggested. Our study reiterates the importance of keeping in mind the assumptions of the methods used and explicitly testing the conclusions derived from population genetic analyses in order to prevent overinterpreting the obtained results and selecting the appropriate analyses (and datasets) for the question at hand.

## Author Contributions

**Carlo Pacioni:** conceptualization (lead), data curation (lead), formal analysis (lead), methodology (lead), validation (lead), writing – original draft (lead), writing – review and editing (lead). **Danielle Stephens:** data curation (supporting), visualization (supporting), writing – review and editing (supporting).

## Conflicts of Interest

The authors declare no conflicts of interest.

## Data Availability

All the data used in this study are openly available from published studies.
